# Diversity and distribution of bacterial phytases

**DOI:** 10.1002/imo2.70104

**Published:** 2026-05-21

**Authors:** Michael Weber, Lena‐Sophie Paul, Thilo M. Fuchs

**Affiliations:** ^1^ Friedrich‐Loeffler‐Institut Institute of Molecular Pathogenesis Jena Germany

## Abstract

**Phytases:** The stepwise dephosphorylation of phytate is catalyzed by phytases, a group of enzymes found mainly among bacteria and plants. **Bacterial potential:** We identified an unexpectedly widespread and previously underappreciated bacterial potential for phytate dephosphorylation, suggesting that microbial phytases play crucial roles in the environmental and host‐associated phosphorus flux. **Diversity:** We analyzed 115,379 validated bacterial genomes to resolve the taxonomic distribution and diversity of potentially phytase‐producing bacterial species, and identified phytase genes in 6281 out of 25,105 species (25%). **Prevalence:** Phytases were predominantly found in members of the phyla Pseudomonadota, Bacteroidota, and Cyanobacteriota, but were comparatively rare in Bacillota and Actinomycetota, with alkaline β‐propeller phytases as the most prevalent and widespread category.

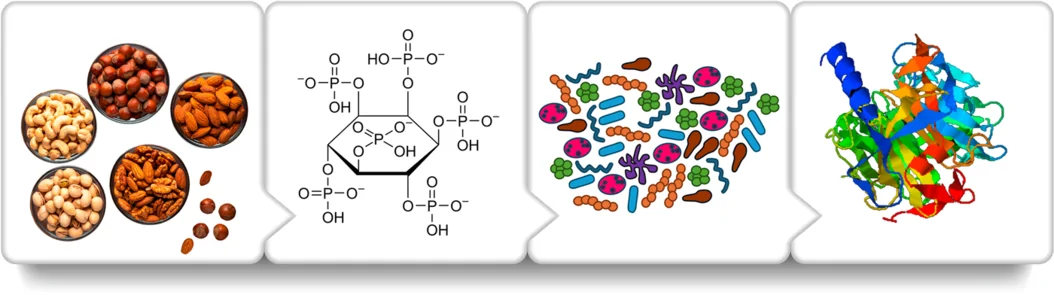

To the Editor,

Phytate, or inositol hexakisphosphate [InsP_6_], is the primary storage form of phosphorus and minerals in plants. It is particularly present in plant tissues such as bran and seeds of legumes and cereals, oil seeds, and nuts. Phytate and other inositol phosphates account for up to 65–80% of the total phosphorus in seed and for up to 80% of total phosphorus in the manure of monogastric animals [1]. Phytate‐hydrolyzing phosphatases, termed phytases, release phosphorus during food processing and in the gastrointestinal tract, thereby increasing the bioavailability of phosphorus and helping to mitigate environmental eutrophication [2]. Additionally, phytases catalyze phosphate release in soil, where phytate represents the most recalcitrant phosphorus‐containing compound [3]. The stepwise hydrolysis of phytate produces *myo*‐Ins phosphate esters (IP_1_ − IP_5_), ultimately yielding free *myo*‐Ins [4]. In the stomach and small intestine of humans, phytases are estimated to hydrolyze 37–66% of all dietary phytate when a diet is rich in plant‐based foods containing active phytases [5]. This contrasts with Western diets that are typically poor in plant food and often lack active phytases due to heating during food processing.

Phytases are mainly of plant (endogenous) or microbial (exogenous) origin. Because plant‐derived phytases do not fully hydrolyze phytate, microbial phytases are frequently added to animal feed, particularly for monogastric species, to mitigate phytate‐bound phosphate limitations in animal production [6]. Alternatively, inoculation with phytase^+^ strains into gastrointestinal tracts of animals has been proposed, though concerns have been raised regarding colonization stability and potential environmental contamination through fecal shedding by those probiotics [6]. Food supplementation can decrease fecal phosphorus to below 1% [7], a significant reduction given that a single pig excretes 1.23 kg of phosphorus over its lifetime in the United States [6]. Dephosphorylation of phytate not only enhances phosphorus bioavailability in livestock [8], but also that of essential dietary minerals [9], as phosphate groups increase the dietary zinc and iron binding capacity of phytate. On the other hand, such an intervention in human nutrition remains controversial because phytate acts as an antioxidant of food components by chelating free iron, thus preventing iron‐driven hydroxyl radical formation [10].

Phytases are commonly classified by their optimal pH, their three‐dimensional structures, and their catalytic mechanisms into categories such as histidine acid phytases (HAPhys), alkaline β‐propeller phytases (BPPhys), and protein tyrosine phytases (PTPhys) [6]. All three groups are produced by bacteria [6], whereas purple acid phytases (PAPhys) are plant‐specific. The multiple inositol polyphosphate phosphatase (MINPP), a subclass of the HAPhy family, has been found in plants and animals [11, 12]. Cysteine phytases, mainly produced by ruminal bacteria, belong to the PTPhy category [13]. Phytate‐degrading microorganisms have been isolated from diverse environments, including marine ecosystems, soils, and the gastrointestinal tract of animals [14, 15].

Despite the critical roles of phytate and phytases in human and animal health and in the global phosphorus cycle, the distribution of bacterial phytases is largely unknown. Since a pilot bioinformatic study from 2007 was based on just 80 bacterial genomes [16], a comprehensive genome‐based bioinformatic approach was performed to identify phytase gene‐positive bacteria across all phyla.

## 1 IN SILICO CLUSTERING OF PHYTASE GENES

To investigate the presence and distribution of phytase genes in bacteria, we performed a comprehensive data mining approach and compiled a set of 34 non‐redundant amino acid sequences of bacterial phytases with experimentally verified phytate‐dephosphorylating activity in the literature (Table S1). These phytases originate from 28 distinct bacterial species, as well as four uncultured and uncharacterized microorganisms [13, 17]. To identify phytase orthologs, we queried the 34 protein sequences against the NCBI non‐redundant protein database. In total, we screened 115,379 bacterial genome assemblies representing 25,105 species, for homologs exhibiting ≥20% sequence identity (e‐value ≤ 10^−06^) and 50% query coverage to at least one of the 34 phytases. A species was classified as phytase‐positive (phytase^+^) if at least one genome within that species encoded a phytase homolog. Overall, we identified putative phytases in 6281 bacterial species, corresponding to approximately 25% of all species examined (Table S2).

To explore the clustering of phytase types, we calculated the Jaccard coefficient for all pairwise BLAST hit combinations among the 34 reference phytases. This analysis identified five clusters, while three enzymes appeared unique within the reference set. The largest cluster included 15 HAPhys represented by the bifunctional 4‐phytase AppA from *E. coli*. A taxonomic analysis showed that HAPhy homologs were present in approximately 7.3% of all species tested here. EEG70762 and ACJ51391 (Table S1) did not exhibit significant similarities to AppA‐like phytases, but were identified as histidine‐phosphatases (HPs) based on BLAST analysis. Homologs of these two HPs were detected in 3.5% of all 25,105 species. Overlap between the two HAPhy groups was rarely found, as of the 1824 and 884, respectively, species harboring the two groups, only 10 species, including 6 *Gilliamella* strains, a honey bee gut symbiont, encoded both types (Table S2). This limited co‐occurrence suggests that AppA‐like phytase and the two HPs may serve similar functions. Given their potential relevance as a novel subgroup of phytase‐like enzymes, we concluded HPs in subsequent analyses. We also identified bacterial homologs of MINPPs classified as a subclass of HAPhys [12]. For all but two of the 34 phytases, a signal peptide sequence was predicted with a probability >0.75, indicating that these enzymes are likely secreted (Table S1).

The second largest cluster consisted of BPPhys, a group of alkaline phosphatases represented by PhyC from *Bacillus amyloliquefaciens*. BPPhy homologs were present in 14.7% of all species screened. Four PTPhys, homologous to the *Selenomonas ruminantium* alkaline phosphatase, formed another distinct cluster, with homologs identified in 1.2% (308) of all species.

Five of the identified phytases (AKS25181, AAC05186, ADX99261, ADY15023, and ADY15024) exhibited homology to proteins with unspecific or poorly characterized annotations. AKS25181 is related to a protein tyrosine phosphatase‐like phytase but is distinct from the PTPhy group. AAC05186 and ADX99261 could not be assigned to any of the three major bacterial phytase categories, and ADX99261 was identified in 95.4% of all species analyzed. ADY15023 and ADY15024 showed no significant similarity to known phosphatase families and are members of the widespread major facilitator superfamily [18]. To prevent misattribution, these five proteins were excluded from subsequent analyses.

## 2 MULTIPLE PHYTASES IN BACTERIAL GENOMES

The majority of species (5866) encode (one or more) phytases from a single cluster. In contrast, the genomes of 400 species code for phytases belonging to two distinct clusters, and only 15 species from the genera *Xanthomonas* and *Pseudomonas* carry phytases from three different categories (HAPhy, BPPhy, and PTPhy) (Figure 1A). The largest group among species with phytase from two clusters comprises 254 species that carry both HAPhy‐ and BPPhy‐like phytases. Of these, 196 belong to ubiquitous genera such as *Sphingomonas* (64 species), *Pseudomonas* (50), *Stenotrophomonas* (30), *Xanthomonas* (27), and *Caulobacter* (25), which are commonly associated with soil, plant, or aquatic environments (Table S2). Additionally, 15 species encode both a HAPhy and a HP phytase, while 34 species from the soil‐dwelling genus *Janthinobacterium* carry genes encoding BPPhys as well as HP phytases.

**Figure 1:**
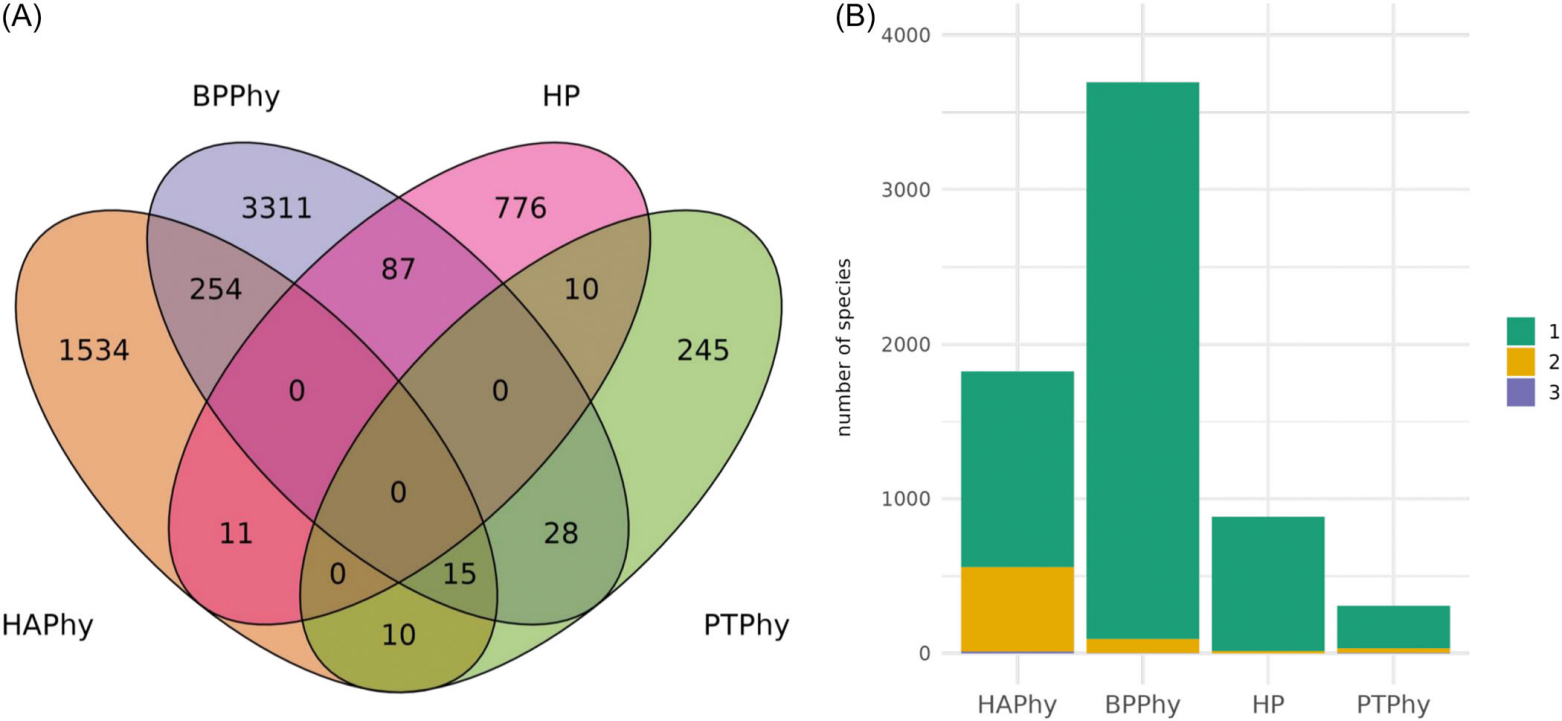
Co‐occurrence of multiple phytases in bacterial genomes. (A) The Venn diagram illustrates the number of species genomes encoding one, two, or three phytases belonging to the four clusters histidine acid phytases (HAPhys), histidine phosphatases (HPs), alkaline β‐propeller phytases (BPPhys), and protein tyrosine phytases (PTPhys). (B) The barplot depicts the number of different phytase clusters per species.

We also analyzed the number of species that harbor more than one phytase gene encoding enzymes from the same cluster (Figure 1B). Among the 1824 species with at least one HAPhy gene, 558 (30.6%) contain two or three homologs, which primarily represent AgpE‐like glucose‐1‐phosphatases and AppA‐like phytases. Similarly, two or three PTPhy genes were found in 10.7% of all species containing PTPhys. The corresponding percentages for BPPhys and HPs were 2.5% and 1.7%. Table S3 shows that a notable subset of bacteria, particularly those from ecologically diverse and plant‐associated and soil‐dwelling genera, possess multiple and phylogenetically distinct phytase types.

## 3 TAXON‐SPECIFIC DISTRIBUTION OF PHYTASES IN BACTERIAL GENOMES

To explore the taxonomic prevalence of phytase genes, we analyzed all 6281 bacterial species identified as phytase^+^ (Table S2, Figure 2A). Among the major phyla Pseudomonadota, Actinomycetota, Bacillota, Bacteroidota, and Cyanobacteriota, and Mycococcota, BPPhys were the most prevalent (Figure 2B). With a focus on families comprising over 100 species, BPPhy‐positive genomes were highly abundant in Xanthomonadaceae (86% phytase^+^ genera), Caulobacteraceae (82%), Sphingobacteriaceae (64%), Erythrobacteraceae (62%), Sphingomonadaceae (61%), Pseudoalteromonadaceae (56%), and Weeksellaceae (53%). AppA‐like HAPhys were predominantly present in Erwiniaceae (98% phytase^+^ genera), Enterobacteriaceae (98%), and Yersiniaceae (96%), all belonging to Pseudomonadota, but largely absent in most other phyla. HP homologs are less widely distributed but were notably enriched in Moraxellaceae (44% phytase^+^ genera), Oxalobacteraceae (32%), Burkholderiaceae (23%), and Bifidobacteriaceae (16%). The less common PTPhys were primarily associated with the phylum Bacillota, especially in Clostridiaceae (30%). To illustrate the distribution of phytases at the genus level, separate cladograms were generated for HAPhys and BPPhys (Figure 2C,D). The HAPhy cladogram encompasses six phyla, 27 families, and 84 genera, while the BPPhy cladogram depicts five phyla, 26 families, and likewise 84 genera. Details are given in Table S4.

**Figure 2:**
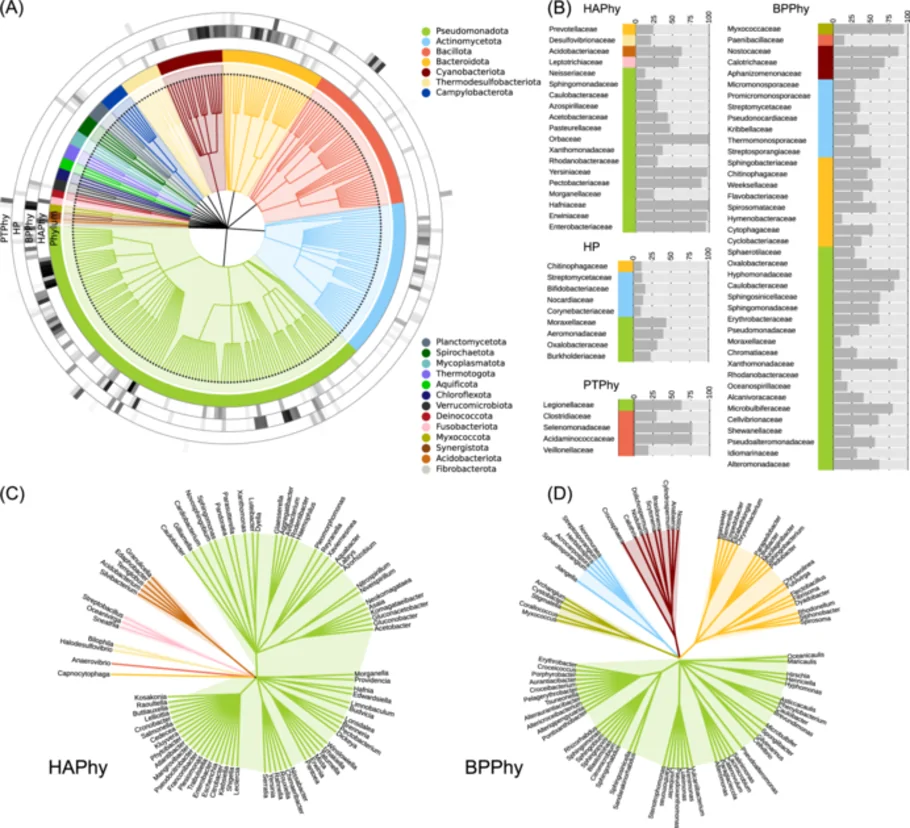
Phytase distribution across the bacterial kingdom. (A) The taxonomic cladogram shows the presence of the four phytase categories in phyla, classes, orders, and families. Tree leaves represent families. Only phyla with more than 25 species and families with at least five species are included. Each outer circle represents one phytase cluster, with a gray‐scale gradient indicating the proportion of phytase^+^ species within each family; darker shades indicate higher prevalence. (B) Phytase cluster barplots display taxonomic families and the relative percentage of species carrying a phytase. Only families comprising at least 10 species and at least 10% prevalence of phytase genes are depicted. The cladograms for the HAPhy (C) and BPPhy (D) clusters show genus‐level relationships based on taxonomy. Only genera with at least 50% phytase^+^ species are shown. Genera represented by a single phytase^+^ species were not considered.

To summarize, phytase genes are broadly distributed throughout bacterial taxa, with BPPhys emerging as the most widespread in diverse phyla and families, while HAPhys, HPs, and PTPhys exhibit a more taxon‐specific enrichment, thus stressing distinct evolutionary patterns and ecological adaptations of bacteria carrying phytase genes.

The large‐scale analysis performed here provides insights into the taxonomic breadth and diversity of phytase‐gene harboring bacteria in all taxa. The extensive catalog of phytase^+^ bacterial species possibly producing phytases serves as a valuable resource for identifying novel phytases, potentially tailored for species and diets, and phytases with novel functions in metabolism and health [6]. Further functional assessment, including heterologous gene expression and metatranscriptomics, however, is needed, extending beyond enzymatic properties to encompass ecological roles and interspecies interactions [14]. Moreover, experimental studies using defined microbial consortia will be essential for uncovering yet unknown synergistic phytase activities within environmental and gut habitats. For example, multiple *myo*‐Ins cross‐feeding was recently observed between a piglet‐derived bacterial consortium of seven phytase^+^ strains to *Brevibacillus agri* and *S. enterica* serovar Typhimurium [19]. Taken together, our findings suggest that an unexpectedly large portion of bacteria may contribute to the metabolization of phytate both in the gut and in biogeochemical systems.

## AUTHOR CONTRIBUTIONS

**Thilo M. Fuchs**: Conceptualization; supervision; writing—original drafts. **Michael Weber**: Bioinformatic analysis; visualization. **Lena‐Sophie Paul**: Data curation; formal analysis. All authors have read the final manuscript and approved it for publication.

## CONFLICT OF INTEREST STATEMENT

The authors declare no conflicts of interest.

## ETHICS STATEMENT

No animals or humans were involved in this study.

## Supporting information


**Table S1:** List of phytases.
**Table S2:** Taxon list of 6,281 species.
**Table S3:** More than 1 phytase per category.
**Table S4:** Phytase gene prevalence across all taxa.

Supporting File

## Data Availability

The data that support the findings of this study are available in the supplementary material of this article. The datasets supporting the conclusions of this article are included within the article and its additional files. The data used are saved in GitHub https://github.com/mweberr/phytases-data. Supplementary materials (methods, tables, graphical abstract, slides, videos, Chinese translated version, and updated materials) may be found in the online DOI or iMetaOmics http://www.imeta.science/imetaomics/.

## References

[1] He, Zhongqi , Barbara J. Cade‐Menun , Gurpal S. Toor , Ann‐Marie Fortuna , C. Wayne Honeycutt , and J. Thomas Sims . 2007. “Comparison of Phosphorus Forms in Wet and Dried Animal Manures by Solution Phosphorus‐31 Nuclear Magnetic Resonance Spectroscopy and Enzymatic Hydrolysis.” Journal of Environmental Quality 36: 1086–1095. 10.2134/jeq2006.0549 17526888

[2] Yuan, Xiao‐Jie , Rui Liu , Jian Li , Wen‐Xiao Zhao , Hui‐Hui Fu , Yan‐Rong Zhou , Mei‐Ling Sun , Xiu‐Lan Chen , and Yu‐Qiang Zhang . 2025. “Diversity Analysis of Phytase‐Producing Bacteria From Coastal Seawater and Sediment and Characterization of Their Phytases.” Engineering Microbiology 5: 100223. 10.1016/j.engmic.2025.100223 PMC12967828 41982653

[3] Yao, Qiuming , Zhou Li , Yang Song , S. Joseph Wright , Xuan Guo , Susannah G. Tringe , and Malak M. Tfaily , et al. 2018. “Community Proteogenomics Reveals the Systemic Impact of Phosphorus Availability on Microbial Functions in Tropical Soil.” Nature Ecology & Evolution 2: 499–509. 10.1038/s41559-017-0463-5 29358607

[4] Kumar, Vikas , Amit K. Sinha , Harinder P. S. Makkar , and Klaus Becker . 2010. “Dietary Roles of Phytate and Phytase in Human Nutrition: A Review.” Food Chemistry 120: 945–959. 10.1016/j.foodchem.2009.11.052

[5] Sandberg, Ann‐Sofie , and Henrik Andersson . 1988. “Effect of Dietary Phytase on the Digestion of Phytate in the Stomach and Small Intestine of Humans.” Journal of Nutrition 118: 469–473. 10.1093/jn/118.4.469 2833590

[6] Lei, Xin Gen , Jeremy D. Weaver , Edward Mullaney , Abul H. Ullah , and Michael J. Azain . 2013. “Phytase, a New Life for an “Old” Enzyme.” Annual Review of Animal Biosciences 1: 283–309. 10.1146/annurev-animal-031412-103717 25387021

[7] Lei, X. G. , P. K. Ku , E. R. Miller , M. T. Yokoyama , and D. E. Ullrey . 1993. “Supplementing Corn‐Soybean Meal Diets With Microbial Phytase Maximizes Phytate Phosphorus Utilization by Weanling Pigs.” Journal of Animal Science 71: 3368–3375. 10.2527/1993.71123368x 8294289

[8] Simons, P. C. M. , H. A. J. Versteegh , A. W. Jongbloed , P. A. Kemme , P. Slump , K. D. Bos , M. G. E. Wolters , R. F. Beudeker , and G. J. Verschoor . 1990. “Improvement of Phosphorus Availability by Microbial Phytase in Broilers and Pigs.” British Journal of Nutrition 64: 525–540. 10.1079/bjn19900052 2171634

[9] Sandberg, Ann‐Sofie , Mats Brune , Nils‐Gunnar Carlsson , Leif Hallberg , Erika Skoglund , and Lena Rossander‐Hulthén . 1999. “Inositol Phosphates With Different Numbers of Phosphate Groups Influence Iron Absorption in Humans.” American Journal of Clinical Nutrition 70: 240–246. 10.1093/ajcn.70.2.240 10426701

[10] Graf, E. , K. L. Empson , and J. W. Eaton . 1987. “Phytic Acid. A Natural Antioxidant.” Journal of Biological Chemistry 262: 11647–11650. 10.1016/S0021-9258(18)60858-0. https://www.ncbi.nlm.nih.gov/pubmed/3040709 3040709

[11] Mullaney, Edward J. , and Abul H. J. Ullah . 1998. “Identification of a Histidine Acid Phosphatase (*phya*)‐like Gene in *Arabidopsis thaliana* .” Biochemical and Biophysical Research Communications 251: 252–255. 10.1006/bbrc.1998.9452 9790941

[12] Stentz, Régis , Samantha Osborne , Nikki Horn , Arthur W. H. Li , Isabelle Hautefort , Roy Bongaerts , Marine Rouyer , et al. 2014. “A Bacterial Homolog of a Eukaryotic Inositol Phosphate Signaling Enzyme Mediates Cross‐Kingdom Dialog in the Mammalian Gut.” Cell Reports 6: 646–656. 10.1016/j.celrep.2014.01.021 PMC3969271 24529702

[13] Huang, Huoqing , Rui Zhang , Dawei Fu , Jianjie Luo , Zhongyuan Li , Huiying Luo , Pengjun Shi , et al. 2011. “Diversity, Abundance and Characterization of Ruminal Cysteine Phytases Suggest Their Important Role in Phytate Degradation.” Environmental Microbiology 13: 747–757. 10.1111/j.1462-2920.2010.02379.x 21105982

[14] Hill, Jane E. , and Alan E. Richardson . 2006. “Isolation and Assessment of Microrganisms that Utilize Phytate.” In Inositol Phosphates‐Linking Agriculture and the Environment, eds B. Turner , A. Richardson and E. Mullaney UK CABI Oxford.

[15] Castillo Villamizar , Genis Andrés , Katrina Funkner , Heiko Nacke , Karolin Foerster , and Rolf Daniel . 2019. “Functional Metagenomics Reveals a New Catalytic Domain, the Metallo‐β‐Lactamase Superfamily Domain, Associated With Phytase Activity.” mSphere 4, no. 3: e00167‐19. 10.1128/mSphere.00167-19 PMC6584368 31217298

[16] Lim, Boon Leong , Pok Yeung , Chiwai Cheng , and Jane Emily Hill . 2007. “Distribution and Diversity of Phytate‐Mineralizing Bacteria.” ISME Journal 1: 321–330. 10.1038/ismej.2007.40 18043643

[17] Tan, Hao , Marlies J. Mooij , Matthieu Barret , Pardraig M. Hegarty , Catriona Harington , Alan D. W. Dobson , and Fergal O'Gara . 2014. “Identification of Novel Phytase Genes From an Agricultural Soil‐Derived Metagenome.” Journal of Microbiology and Biotechnology 24: 113–118. 10.4014/jmb.1307.07007 24150499

[18] Sarikhani, Mohammad Reza , Mohammad Ali Malboobi , Nasser Aliasgharzad , Ralf Greiner . 2019. “Identification of Two Novel Bacterial Phosphatase‐Encoding Genes in *Pseudomonas putida* Strain P13.” Journal of Applied Microbiology 127: 1113–1124. 10.4014/jmb.1307.07007 31287935

[19] Paul, Lena‐Sophie , Michael Weber , Stefanie Wagner , and Thilo M. Fuchs . 2026. “A Culturomics Approach Reveals Cross‐Feeding Capacity of Intestinal Pig Bacteria Upon Release of Inositol From Phytate.” Microbiome 14: 44. 10.1186/s40168-025-02313-5 PMC12838507 41566379

