# Supplementary material for: Diversity and distribution of bacterial phytases
Source: IMetaOmics. 2026 May 21;3(2):e70104. doi: 10.1002/imo2.70104 (PMC13674438; doi:10.1002/imo2.70104)
Supplement: Supplementary file 1 [file IMO2-3-e70104-s001.docx]

Supporting information to

**Diversity and distribution of bacterial phytases**

Running title: Bacterial phytases

Michael Weber^1^, Lena-Sophie Paul^1^, and Thilo M. Fuchs^1*^

^1^Friedrich-Loeffler-Institut, Institute of Molecular Pathogenesis, Naumburger Str. 96a, 07743 Jena, Germany

^*^Correspondence: [thilom.fuchs@fli.de](mailto:thilom.fuchs@fli.de) (Thilo M. Fuchs)

**METHODS**

**Preparation of bacterial protein database.** The nr protein database was downloaded from NCBI in December 2023 using the Perl program update_blastdb, which provides access to the current database via cloud providers. This database contains sequence data from over 365,700 bacterial isolates.

**Selection of genome assemblies.** A comprehensive set of genome assemblies was used to screen for phytase-encoding genes. All assemblies were downloaded from the NCBI ftp server (ftp://ftp.ncbi.nlm.nih.gov/genomes). For each assembly, the nucleotide fasta sequence (FNA), gene annotation file (GFF), and protein sequence file (FAA) were retrieved. Assemblies marked with a refseq exclusion attribute were discarded from the final set (<https://www.ncbi.nlm.nih.gov/assembly/help/anomnotrefseq/>). In cases where gene annotations were missing, Prokka (version 1.14.5) was used with default parameters to annotate protein-coding genes [1].

**Taxonomic analysis.** Taxonomic information was obtained from the NCBI Taxonomy database (http://www.ncbi.nlm.nih.gov/taxonomy). The two central database files, nodes.dmp and names.dmp, were downloaded on January 3, 2024, and processed using the R package taxonomizr (https://github.com/sherrillmix/taxonomizr; Version 0.10.6). These files provide hierarchical taxonomic information, including genus, family, order, class, and phylum for all species analysed. Using the mapping functions in taxonomizr, taxon IDs were assigned to each species and filtered to retain only genomes with complete assignments across all five taxonomic levels. Tree structures were exported in Newick format using the makeNewick function.

**Phytase genome screening.** The 34 phytases sequences from the cluster analysis were used as queries in large-scale, high-throughput genome screens, implemented by a Snakemake annotation pipeline [2]. For each genome, translated protein sequences (FAA) were converted into BLAST databases using makeblastdb. BLASTP searches were performed with a minimum threshold of 20% identity and 50% query coverage. Hits were grouped into four clusters based on the cluster membership of the query sequences. The results were compiled into annotated tables containing information on sequence similarity, coverage, product annotation, and taxonomic classification. Summary tables were generated to assess the prevalence of phytase^+^ species in all taxonomic ranks (phylum, class, order, family, and genus). A Venn diagram, illustrating the intersection of species over the four phytase clusters was created using the R package ggvenn (v. 0.1.10), with species taxon ID lists as input.

**Phylogenie.** Phylogenetic trees were generated with GraPhlAn (version 1.1.4) [3] and ITOL (version 7.2) [4] . All cladograms represent evolutionary relationships inferred from NCBI taxonomy. GraPhlAn tree building required the generation of annotation files as described (https://github.com/biobakery/graphlan). ITOL annotations were customized using template files to modify tree branch and background colours for visual clarity.

**REFERENCES**

[1]	Seemann, Torsten. 2014. Prokka: rapid prokaryotic genome annotation. *Bioinformatics* **30**, 2068˗˗2069, doi:10.1093/bioinformatics/btu153.

[2]	Moelder, Felix, Kim Philipp Jablonski, Brice Letcher, Michael B. Hall,, Peter C. van Dyken , Christopher H. Tomkins-Tinch, Vanessa Sochat, Jan Forster et al*.* 2021. Sustainable data analysis with Snakemake. *F1000Research* **10**, 33, doi:10.12688/f1000research.29032.2.

[3]	Asnicar, Francesco, George, Weingart, Timothy L. Tickle, Curtis. Huttenhower& Nicola Segata. 2025. Compact graphical representation of phylogenetic data and metadata with GraPhlAn. *Peer Journal* **3**, e1029, doi:10.7717/peerj.1029.

[4]	Letunic, Ivica, Tobias Doerks & Peer Bork. 2025. SMART: recent updates, new developments and status in 2015. *Nucleic Acids Research* **43**, D257˗˗260, doi:10.1093/nar/gku949.
